# Individualized assessment predictive models for risk and overall survival in elderly patients of primary kidney cancer with bone metastases: A large population-based study

**DOI:** 10.3389/fmed.2023.1127625

**Published:** 2023-04-25

**Authors:** Liming Jiang, Yuexin Tong, Jiajia Jiang, Dongxu Zhao

**Affiliations:** Department of Orthopedics, The China-Japan Union Hospital of Jilin University, Changchun, Jilin, China

**Keywords:** elderly patients, kidney cancer, bone metastasis, SEER database, nomogram

## Abstract

**Background:**

Elderly people are at high risk of metastatic kidney cancer (KC), and, the bone is one of the most common metastatic sites for metastatic KC. However, studies on diagnostic and prognostic prediction models for bone metastases (BM) in elderly KC patients are still vacant. Therefore, it is necessary to establish new diagnostic and prognostic nomograms.

**Methods:**

We downloaded the data of all KC patients aged more than 65 years during 2010–2015 from the Surveillance, Epidemiology, and End Results (SEER) database. Univariate and multivariate logistic regression analyses were used to study independent risk factors of BM in elderly KC patients. Univariate and multivariate Cox regression analysis for the study of independent prognostic factors in elderly KCBM patients. Survival differences were studied using Kaplan–Meier (K–M) survival analysis. The predictive efficacy and clinical utility of nomograms were assessed by receiver operating characteristic (ROC) curve, the area under curve (AUC), calibration curve, and decision curve analysis (DCA).

**Results:**

A final total of 17,404 elderly KC patients (training set: *n* = 12,184, validation set: *n* = 5,220) were included to study the risk of BM. 394 elderly KCBM patients (training set: *n* = 278, validation set: *n* = 116) were included to study the overall survival (OS). Age, histological type, tumor size, grade, T/N stage and brain/liver/lung metastasis were identified as independent risk factors for developing BM in elderly KC patients. Surgery, lung/liver metastasis and T stage were identified as independent prognostic factors in elderly KCBM patients. The diagnostic nomogram had AUCs of 0.859 and 0.850 in the training and validation sets, respectively. The AUCs of the prognostic nomogram in predicting OS at 12, 24 and 36 months were: training set (0.742, 0.775, 0.787), and validation set (0.721, 0.827, 0.799), respectively. The calibration curve and DCA also showed excellent clinical utility of the two nomograms.

**Conclusion:**

Two new nomograms were constructed and validated to predict the risk of developing BM in elderly KC patients and 12-, 24-, and 36-months OS in elderly KCBM patients. These models can help surgeons provide more comprehensive and personalized clinical management programs for this population.

## Introduction

Kidney cancer (KC) is one of the most common malignant tumors in adults, ranking 6th among male malignant tumors and 9th among female malignant tumors, with a male-to-female ratio of about 1.8:1 (1). Furthermore, the incidence of KC increases gradually with age, reaching a peak at the age of 60–70 years (2). In 2020, the global number of new KC patients is 431,288 and the number of deaths is 179,368, with a mortality rate of 41.6% (3). Patients over 65 years of age accounted for more than 70% of the new patients (4). As medical technology continues to advance and life expectancy is increasing, the number of people over 60 years of age will reach 2.1 billion by 2050, making it particularly essential to optimize the clinical management of elderly KC patients (5, 6).

With the enhancement of medical diagnostic technology, it has enabled many KC patients to be diagnosed early, approximately 20–30% of patients have distant metastases at the time of initial diagnosis (7). Bone is the second most common site of metastasis for metastatic KC (8, 9). Patients of KC with bone metastases (KCBM) tend to have a poor prognosis, with a median survival of only 10.2–13.8 months (10, 11). The probability of skeletal-related events after BM is as high as 74%, which often brings about a range of serious complications, such as pain, pathological fractures, and spinal cord/nerve root compression (12, 13). These complications have serious impacts on patients’ quality of life and overall survival (OS).

Nomogram is a comprehensive prediction model that combines clinical characteristics of patients and is widely used for diagnosis and OS prediction of various tumor diseases due to its simplicity and ease of use (14, 15). Some previous studies have constructed nomograms on the risk of developing BM from KC patients and the prediction of OS for the KCBM patients, but the subjects of these studies have mainly focused on the whole group of KC patients, not the special elderly patients (11, 16). According to our knowledge, studies on predicting the risk of developing BM in elderly KC patients and the OS of elderly KCBM patients are still vacant.

Based on the above factors, we constructed and validated two nomograms on the risk of BM in elderly KC patients and the OS of elderly KCBM patients. In addition, two online versions of the nomograms were generated. The aim is to provide individualized prediction the risk of BM in elderly KC patients and the OS of elderly KCBM patients, and to optimize the clinical management of these patients.

## Methods

### Study population

**Figure 1:**
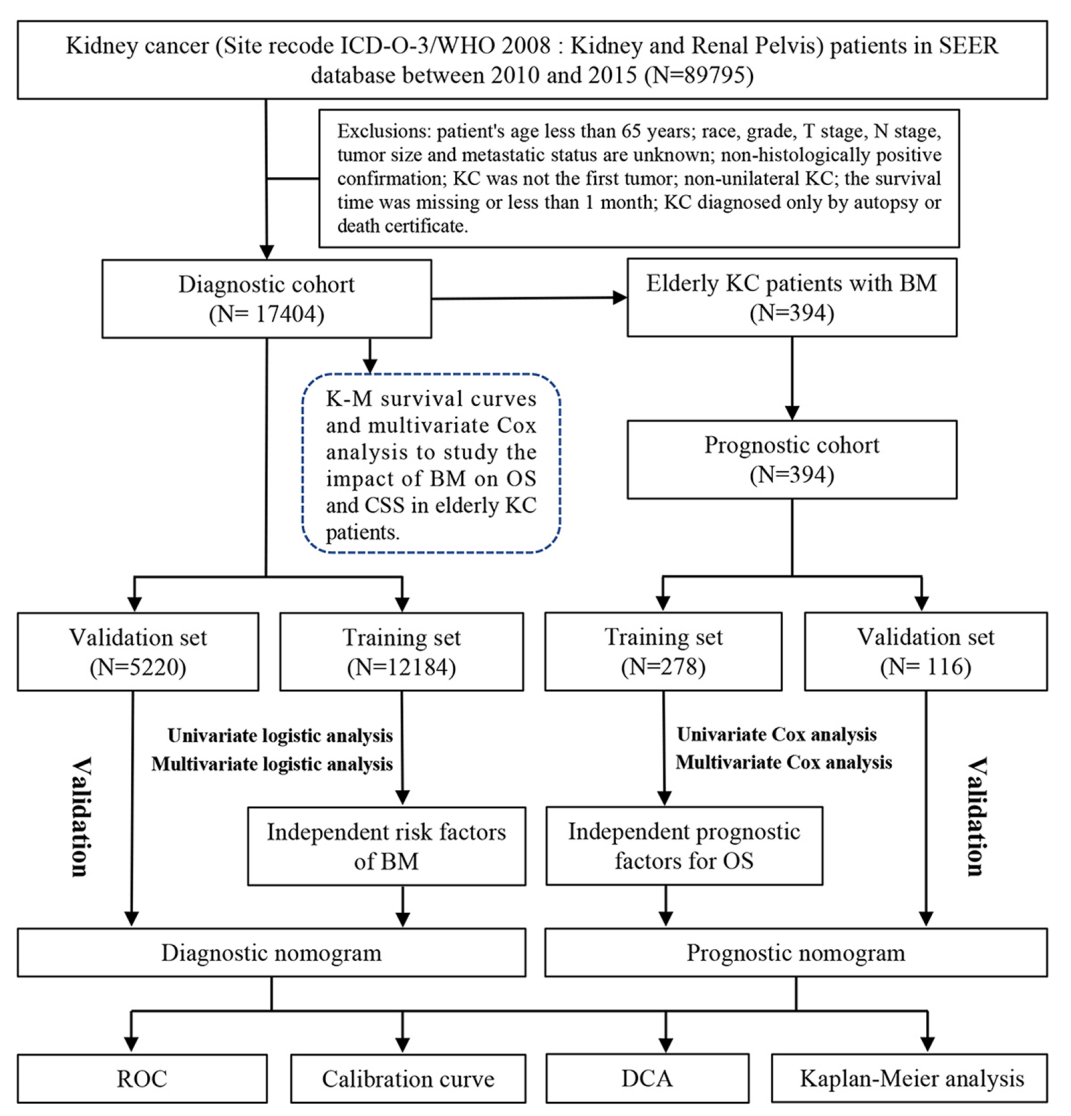
Patients’ selection and workflow of this study.

All of our data were downloaded from the National Cancer Institute’s Surveillance, Epidemiology, and End Results (SEER) database *via* SEER Stat 8.4.1.^1^ The database is currently the largest and publicly available cancer database, containing 18 cancer registries covering approximately 28% of the United States population (17). The SEER database started recording complete cancer metastasis sites from 2010 and for patients to have sufficient follow-up time records, so we selected all KC patient data from 2010 to 2015. A diagnostic cohort of 17,404 elderly KC patients and a prognostic cohort of 394 elderly KCBM patients were finalized by inclusion and exclusion criteria. The inclusion criteria were: (1) KC confirmed by Site recode ICD-O-3/WHO 2008 (Kidney and Renal Pelvis); (2) the time of diagnosis was between 2010 and 2015. Exclusion criteria were (1) patient’s age less than 65 years; (2) race, grade, T-stage, N-stage, tumor size, and metastatic status unknown; (3) non-histologically positive confirmation; (4) KC not being the first primary tumor; (5) non-unilateral KC; (6) missing or less than 1 month of survival; (7) KC diagnosed only by autopsy or death certificate. In addition, the SEER database does not record patient identification information and has been de-identified, so the study did not require ethics committee review and approval. The patient selection and workflow for this study is shown in Figure 1.

### Data selection

We selected 12 variables to study the risk of BM in elderly KC patients: race, sex, age at diagnosis, histological type, laterality of tumor, tumor size, grade, T stage, N stage, brain metastasis, liver metastasis and lung metastasis. Classification variables included: age (<70 years, 70–80 years, >80 years) (18), tumor size (<40 mm, 40-80 mm, >80 mm) (19), grade (I: well differentiated, II: moderately differentiated, III: poorly differentiated, IV: undifferentiated), Laterality (Left and Right). The type of histology was divided into seven categories using the International Classification of Diseases for Oncology, third edition (ICD-O-3) Hist/behav: transitional cell carcinoma (8,120/3), papillary transitional cell carcinoma (8,130/3), papillary adenocarcinoma (8,260/3), clear cell adenocarcinoma (8,310/3), renal cell carcinoma (8,312/3), chromophobe cell carcinoma (8,317/3) and other (not otherwise specified). To study the prognostic factors affecting elderly KCBM patients, we also downloaded treatment information and survival information for these patients, including: surgery, chemotherapy (CT), radiotherapy (RT), OS and cancer-specific survival (CSS). The surgical procedures were classified as: No surgery (SEER codes 0), local excision of tumor (SEER codes 10–27), partial nephrectomy (SEER codes 30), and radical nephrectomy (SEER codes 40–80). OS is the survival time of a patient from the time of diagnosis until death regardless of cause, and CSS is the survival time of a patient from the time of diagnosis until death due to KC alone. Tumor staging was determined according to the American Joint Committee on Cancer (AJCC) 7th TNM.

### Statistical analyses

All statistical analyses were performed with SPSS (version 27.0) and R software (version 4.1.2; https://www.r-project.org/). Firstly, we performed the K–M survival analysis of the overall diagnostic cohort to assess the prognostic impact of BM on elderly KC patients using OS and CSS as survival outcomes. Further assessment of whether BM is an independent prognostic factor in elderly KC patients was performed by univariate and multivariate Cox regression analysis. Then, we randomly divided the overall diagnostic and prognostic cohorts into training and validation sets in a ratio of 7:3 using R software, and the differences of baseline data between sets were tested using Chi-square (× 2) tests (20). Univariate and multivariate logistic regression analyses were performed on the training set of the diagnostic cohort to identify independent risk factors influencing the development of BM in elderly KC patients. In parallel, we also calculated odds ratio (OR) and 95% confidence interval (CI), to show the correlation between different baseline variables and BM development. Similarly, we performed univariate Cox regression analysis for the training set of prognostic cohort, and variables with *p* < 0.05 were included in the multivariate Cox regression analysis to identify independent prognostic factors for elderly patients with KCBM. The effects of different independent prognostic factors on OS are illustrated with hazard rate (HR) and 95% CI (21). The *p* value < 0.05 (95% CI) was considered statistically difference (22).

Subsequently, based on the identified independent risk factors and independent prognostic factors, diagnostic and prognostic nomograms and the corresponding online validation versions were constructed using the “rms” and “DynNom” packages in the R software, respectively. Receiver operating characteristic (ROC) curve, the area under curve (AUC), calibration curve, and decision curve analysis (DCA) were used to assess and analyze the predictive efficacy and clinical utility of the nomograms. By using the constructed prognostic nomogram, we calculated the prognostic score for each elderly KCBM patient. In addition, we utilized the X-Tile software to figure out the optimal cut-off values for each patient’s nomogram total score. Based on these thresholds, we divided the patients into high, medium and low risk groups, and performed K–M survival analysis to assess the efficacy of risk stratification of the prognostic nomogram. In addition, we use time-dependent ROC to compare the predictive efficacy of prognostic nomogram with AJCC TNM staging system.

## Results

### Clinicopathological characteristics of elderly KC patients

**Figure 2:**
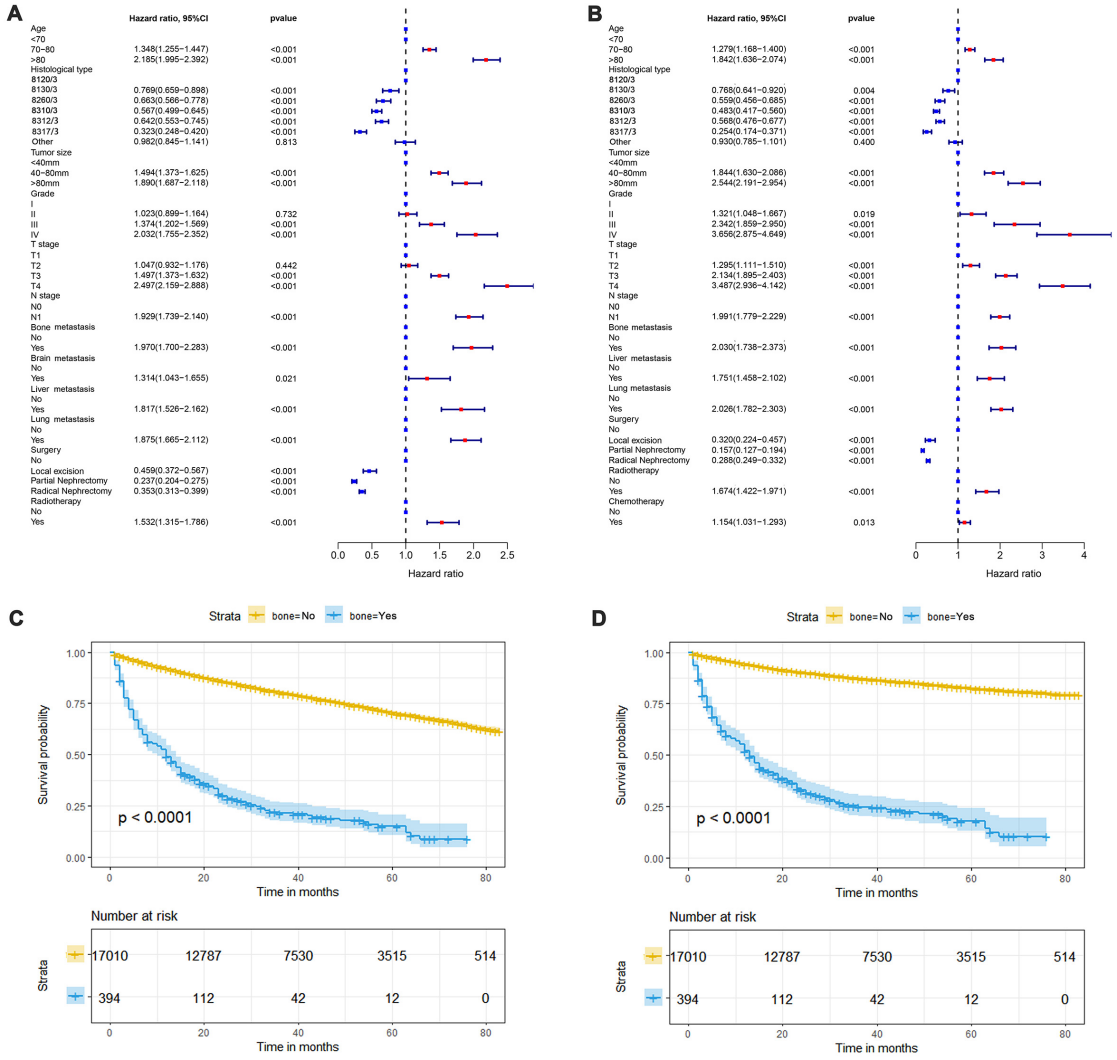
The forest plot shows the results of multivariate Cox regression analysis in which bone metastasis (BM) was significantly associated with overall survival (OS) and cancer-specific survival (CSS) in elderly KC patients **(A,B)**. Kaplan–Meier survival analysis was performed to investigate the effect of BM on OS and CSS in elderly KC patients **(C,D)**.

In our study, 17,404 elderly KC patients were finally recruited. Table 1 records the demographic information and clinicopathological characteristics of these patients, of whom there were more male patients (*n* = 10,522, 60.5%) than female patients (*n* = 6,882, 39.5%) in a ratio of approximately 1.53:1 (Table 1). The age concentration was 65–70 years (*n* = 6,798, 39.1%) and 70–80 years (*n* = 8,452, 48.6%), with relatively few patients over 80 years of age (*n* = 2,154, 12.4%). Race was predominantly white (*n* = 14,660, 84.2%). The histological type was mostly clear cell adenocarcinoma 8310/3 (*n* = 10,570, 60.7%). In terms of tumor size, the majority of patients had tumors ≤ 80 mm in size (< 40 mm: *n* = 7,012, 40.3% and 40–80 mm: *n* = 7,612, 43.7%). Regarding the laterality of the tumors, the number of patients on the left (*n* = 8,645, 49.7%) and right (*n* = 8,759, 50.3%) was comparable. *p* values between all baseline variables were greater than 0.05, indicating that the deviations between baseline variables were completely random (Table 2). As shown in Figure 2, the multivariate Cox regression analysis further illustrated that BM was significantly associated with the OS and CSS of elderly KC patients (Figures 2A,B). Moreover, the elderly KC patients with BM had a worse survival prognosis than those without BM, both in terms of OS and CSS as survival outcomes (*p* < 0.05; Figures 2C,D).

**Table 1:** The demographic and clinicopathological characteristics of the elderly kidney cancer (KC) patients with or without bone metastases (BM).

Variables	Total set (*N* = 17,404,%)	Training set (*N* = 12,184,%)	Validation set (*N* = 5,220,%)	*p* value
Age				0.895
<70	6,798(39.1)	4,772(39.2)	2026(38.8)	
70–80	8,452(48.6)	5,910(48.5)	2,542(48.7)	
>80	2,154(12.4)	1,502(12.3)	652(12.5)	
Sex				0.475
Female	6,882(39.5)	4,839(39.7)	2043(39.1)	
Male	10,522(60.5)	7,345(60.3)	3,177(60.9)	
Race				0.711
Black	1,568(9.0)	1,112(9.1)	456(8.7)	
Other	1,176(6.8)	822(6.7)	354(6.8)	
White	14,660(84.2)	10,250(84.1)	4,410(84.5)	
Histological type				0.649
8120/3	692(4.0)	481(3.9)	211(4.0)	
8130/3	775(4.5)	531(4.4)	244(4.7)	
8260/3	2042(11.7)	1,417(11.6)	625(12.0)	
8310/3	10,570(60.7)	7,386(60.6)	3,184(61.0)	
8312/3	1822(10.5)	1,306(10.7)	516(9.9)	
8317/3	639(3.7)	454(3.7)	185(3.5)	
Other	864(5.0)	609(5.0)	255(4.9)	
Laterality				0.971
Left	8,645(49.7)	6,051(49.7)	2,594(49.7)	
Right	8,759(50.3)	6,133(50.3)	2,626(50.3)	
Tumor size				0.210
≤40 mm	7,012(40.3)	4,948(40.6)	2064(39.5)	
40-80 mm	7,612(43.7)	5,276(43.3)	2,336(44.8)	
≥80 mm	2,780(16.0)	1960(16.1)	820(15.7)	
Grade				0.625
Grade I	1770(10.2)	1,254(10.3)	516(9.9)	
Grade II	8,105(46.6)	5,640(46.3)	2,465(47.2)	
Grade III	5,323(30.6)	3,749(30.8)	1,574(30.2)	
Grade IV	2,206(12.7)	1,541(12.6)	665(12.7)	
T stage				0.467
T1	10,722(61.6)	7,510(61.6)	3,212(61.5)	
T2	1809(10.4)	1,274(10.5)	535(10.2)	
T3	4,426(25.4)	3,102(25.5)	1,324(25.4)	
T4	447(2.6)	298(2.4)	149(2.9)	
N stage				0.542
N0	16,639(95.6)	11,656(95.7)	4,983(95.5)	
N1	765(4.4)	528(4.3)	237(4.5)	
Brain metastasis				0.985
No	17,287(99.3)	12,102(99.3)	5,185(99.3)	
Yes	117(0.7)	82(0.7)	35(0.7)	
Liver metastasis				0.913
No	17,215(98.9)	12,051(98.9)	5,164(98.9)	
Yes	189(1.1)	133(1.1)	56(1.1)	
Lung metastasis				0.225
No	16,689(95.9)	11,698(96.0)	4,991(95.6)	
Yes	715(4.1)	486(4.0)	229(4.4)	

**Table 2:** Univariate and multivariate logistic analysis to determine the independent risk factors of BM in elderly KC patients.

Variables	Univariate analysis			Multivariate analysis		
	HR	95%CIs	*p*-Value	HR	95%CIs	*p*-value
Age						
<70	Reference			Reference		
70–80	0.850	0.663–1.089	0.199	0.853	0.652–1.115	0.245
>80	0.532	0.333–0.847	0.008	0.539	0.327–0.886	0.015
Sex						
Female	Reference					
Male	1.058	0.828–1.352	0.653			
Race						
Black	Reference					
Other	1.360	0.676–2.735	0.389	1.097	0.518–2.323	0.808
White	1.670	1.003–2.781	0.048	1.394	0.806–2.411	0.235
Histological type						
8120/3	Reference			Reference		
8130/3	0.447	0.166–1.200	0.110	1.075	0.382–3.022	0.891
8260/3	0.250	0.105–0.597	0.002	0.892	0.347–2.293	0.813
8310/3	0.832	0.459–1.509	0.545	2.172	1.090–4.325	0.027
8312/3	1.816	0.967–3.412	0.064	3.813	1.859–7.824	<0.001
8317/3	0.173	0.038–0.777	0.022	0.603	0.127–2.861	0.524
Other	2.383	1.223–4.643	0.011	2.791	1.336–5.830	0.006
Laterality						
Left	Reference					
Right	0.930	0.732–1.180	0.548			
Tumor size						
<40 mm	Reference			Reference		
40-80 mm	4.509	2.993–6.792	<0.001	2.425	1.557–3.779	<0.001
>80 mm	11.054	7.291–16.758	<0.001	1.989	1.174–3.371	0.011
Grade						
I	Reference					
II	4.938	1.550–15.729	0.007	4.449	1.363–14.518	0.013
III	14.145	4.492–44.545	<0.001	8.104	2.473–25.971	0.001
IV	24.041	7.581–76.238	<0.001	9.301	2.815–30.730	<0.001
T Stage						
T1	Reference			Reference		
T2	3.731	2.528–5.507	<0.001	1.783	1.126–2.824	0.014
T3	4.903	3.649–6.587	<0.001	1.812	1.256–2.615	0.001
T4	13.629	8.835–21.024	<0.001	2.666	1.500–4.737	0.001
N Stage						
N0	Reference			Reference		
N1	9.830	7.434–12.997	<0.001	2.643	1.874–3.728	<0.001
Brain organ metastasis						
No	Reference			Reference		
Yes	22.011	13.597–35.632	<0.001	5.115	2.896–9.035	<0.001
Liver organ metastasis						
No	Reference			Reference		
Yes	16.759	11.123–25.249	<0.001	3.231	1.991–5.244	<0.001
Lung organ metastasis						
No	Reference			Reference		
Yes	14.890	11.377–19.488	<0.001	3.580	2.566–4.993	<0.001

### Risk factors in development of BM and diagnostic nomogram

**Figure 3:**
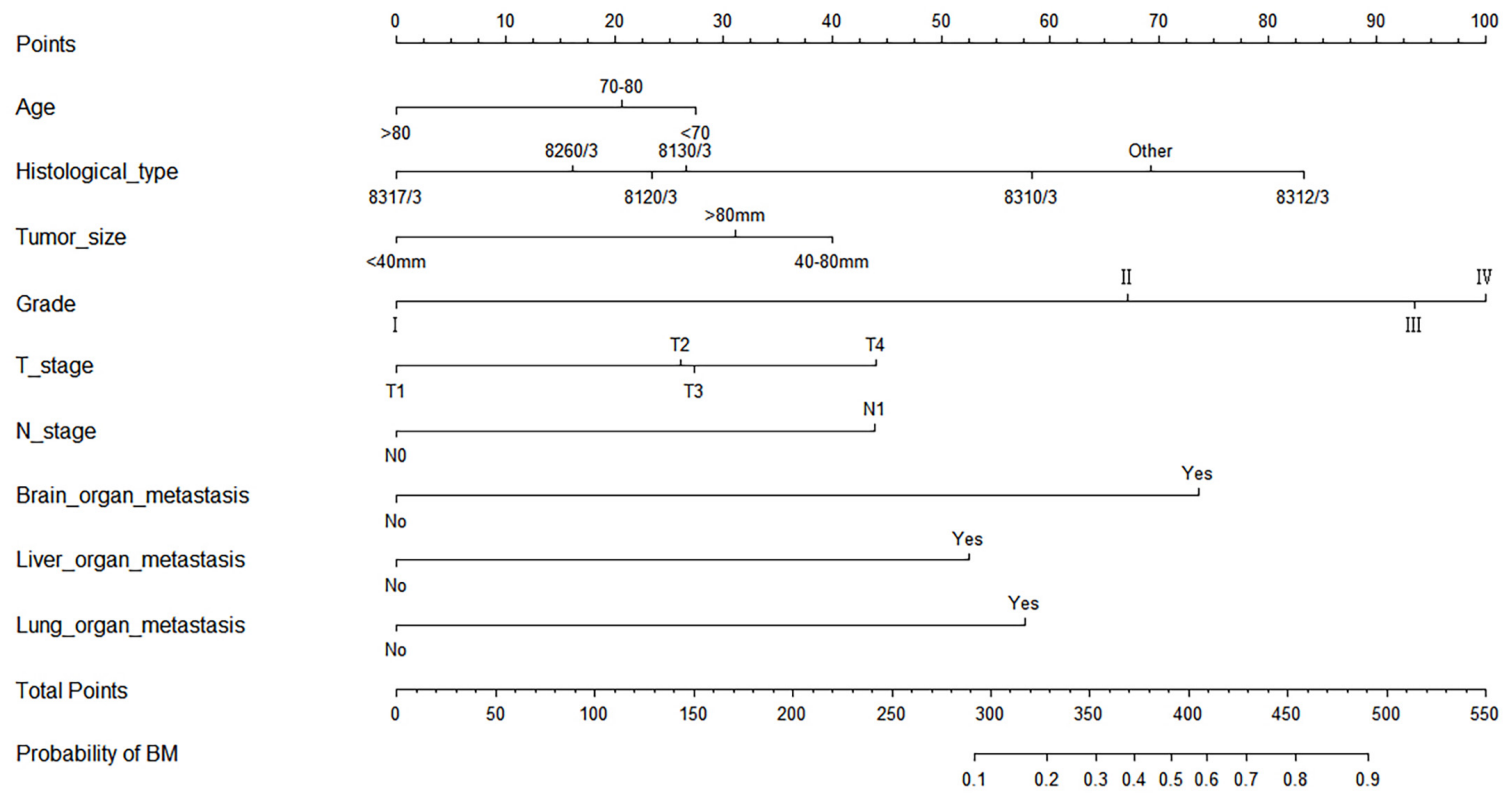
Diagnostic nomogram for quantifying the probability of bone metastasis (BM) in elderly kidney cancer (KC) patients.

**Figure 4:**
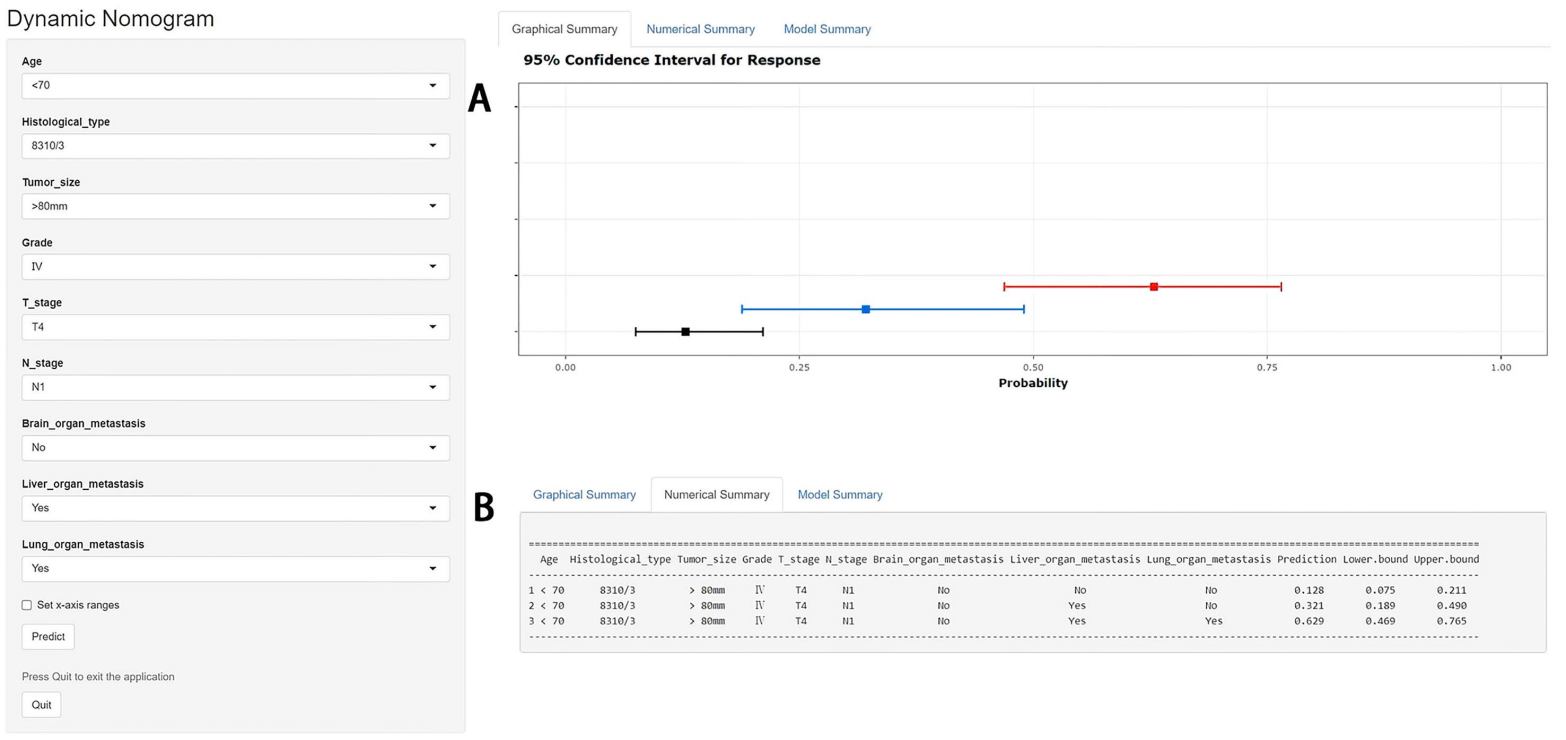
A web-based nomogram for predicting bone metastasis (BM) in an elderly kidney cancer (KC) patient (age: <70, histological type: 8310/3, tumor size: >80 mm, grade: IV, T stage: T4, N stage: N1). 95% confidence intervals of without brain/liver/lung metastasis, with liver metastasis and with liver/lung metastasis BM probabilities for this patient **(A)**. Numerical summary of without brain/liver/lung metastasis, with liver metastasis and with liver/lung metastasis BM probabilities for this patient **(B)**. Due to a large number of visitors to the webpage, if the application cannot be used normally, please click “Quilt” or “Reload” in the lower-left corner to try again.

As shown in Table 2, the results of univariate and multivariate logistic regression analysis showed that age, histological type, tumor size, grade, T stage, N stage, brain/ liver and lung metastasis were independent risk factors for the development of BM in elderly KC patients (Table 2). Briefly, in KC patients over 65 years of age, the younger the age and the higher the grade and T/N stage of tumor, the greater the risk of developing BM. Elderly KC patients with histological type of renal cell carcinoma (8,312/3), tumor size of 40–80 mm, and brain/ liver/ lung metastases were at higher risk of developing BM. Based on these nine independent risk factors, a diagnostic nomogram was constructed to quantify the risk of BM (Figure 3), and an online version can be accessed through https://jianglim.shinyapps.io/dynnomapp/ (Figure 4).

**Figure 5:**
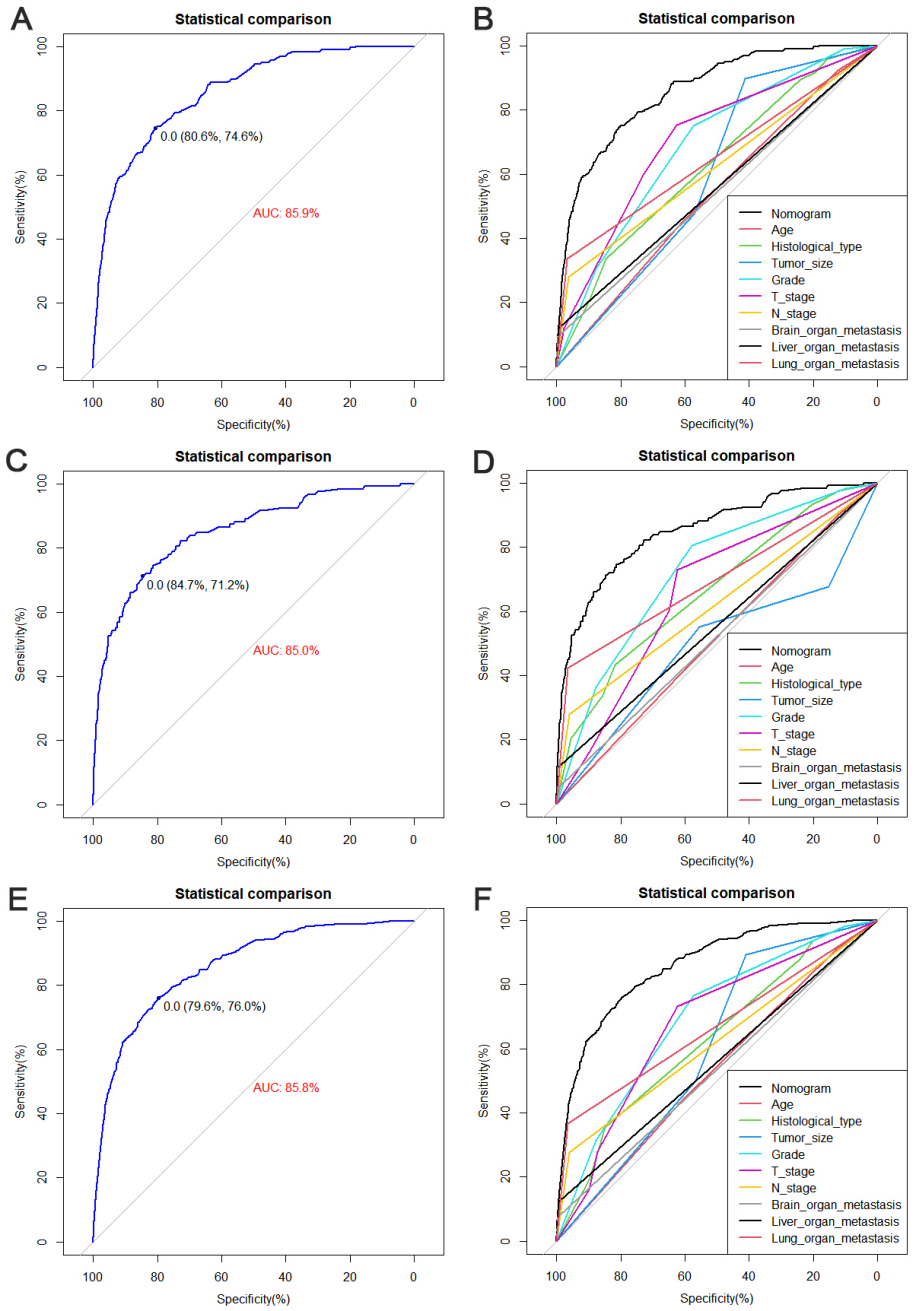
Receiver operating characteristic (ROC) curves for the training set **(A)**, the validation set **(C)**, and the extended validation set **(E)**. Comparison of nomograms and the area under curve (AUC) of all predictors in the training set **(B)**, validation set **(D)**, and extended validation set **(F)**.

**Figure 6:**
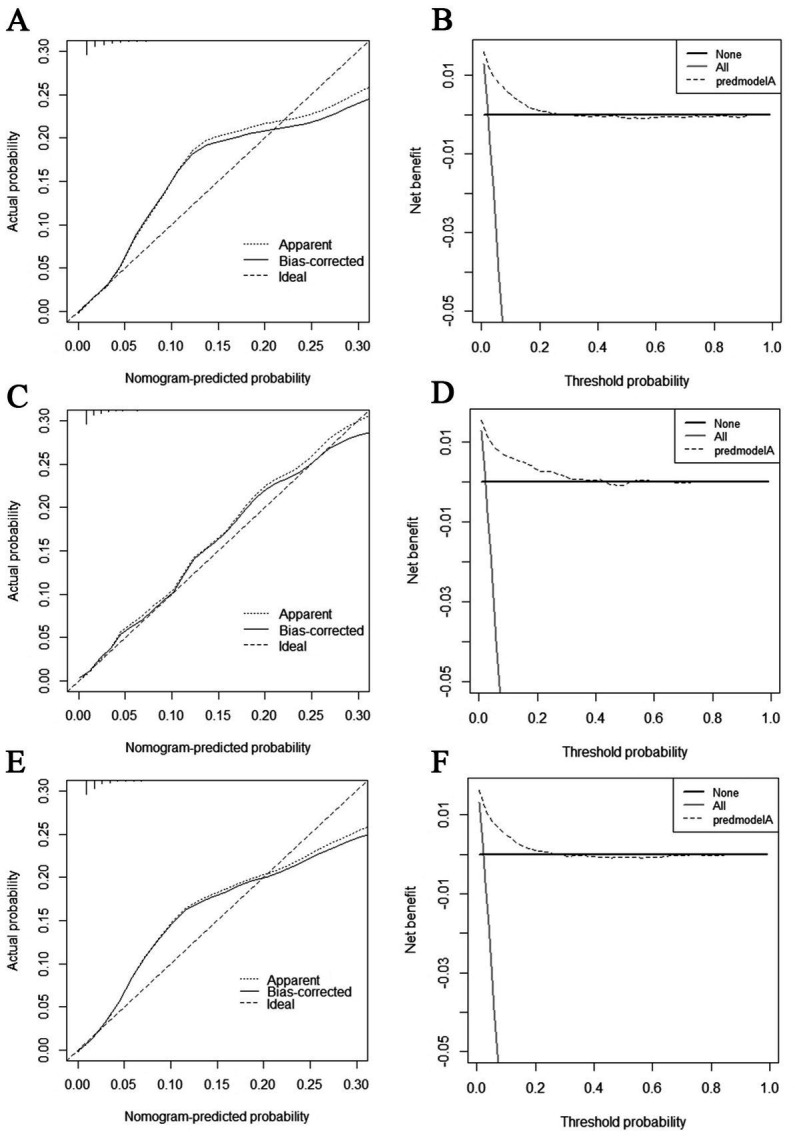
Calibration curves and decision curve analysis (DCA) curves for the training set **(A,B)**, calibration curves and DCA curves for the validation set **(C,D)**, and calibration curves and DCA curves for the extended validation set **(E,F)**.

The diagnostic nomogram produced an AUC value of 0.859 (95% CI: 0.837–0.880) in the training set and 0.850 (95% CI: 0.812–0.889) in the validation set, indicating that the nomogram has excellent predictive efficacy (Figures 5A,C). Next, we compared the predictive efficacy of diagnostic nomogram and single independent risk factors using ROC curves, and the AUCs of single independent risk factors were all smaller than the nomogram (*p* < 0.05; Figures 5B,D). Furthermore, the calibration curves and DCA showed that the predicted probability of the nomogram were in good agreement with the actual probability and had a high net benefit, which implies that the diagnostic nomogram have good clinical utility for predicting the development of BM in elderly KC patients (Figures 6A–D). However, due to the low prevalence of BM in the elderly KC patients, 2.3% in the present study, it was rather challenging to find a sufficient number of external validation sets in the same area. Therefore, we returned to the database to re-screen 18,274 elderly KC patients as an extended validation set for this study by the nine independent risk factors identified. The inclusion criteria were: (1) KC confirmed by Site recode ICD-O-3/WHO 2008 (Kidney and Renal Pelvis); (2) the time of diagnosis was between 2010 and 2015; (3) patients were 65 years of age and older; (4) KC was the first malignant primary indicator; (5) KC was diagnosed by histologically positive confirmation. The exclusion criteria were: (1) patient’s tumor size, grade and T/N stage were unknown; (2) the bone/ brain/liver/lung metastatic status was unknown. In the extended validation set, the nomogram yielded an AUC value of 0.858 (95% CI: 0.840–0.875; Figure 5E). The ROC comparison curves, calibration curves, and DCA of the extended validation set demonstrate the excellent predictive efficacy and clinical utility of the diagnostic nomogram (Figure 5F; Figures 6E,F).

### Prognostic factors and nomogram in elderly KCBM patients

**Figure 7:**
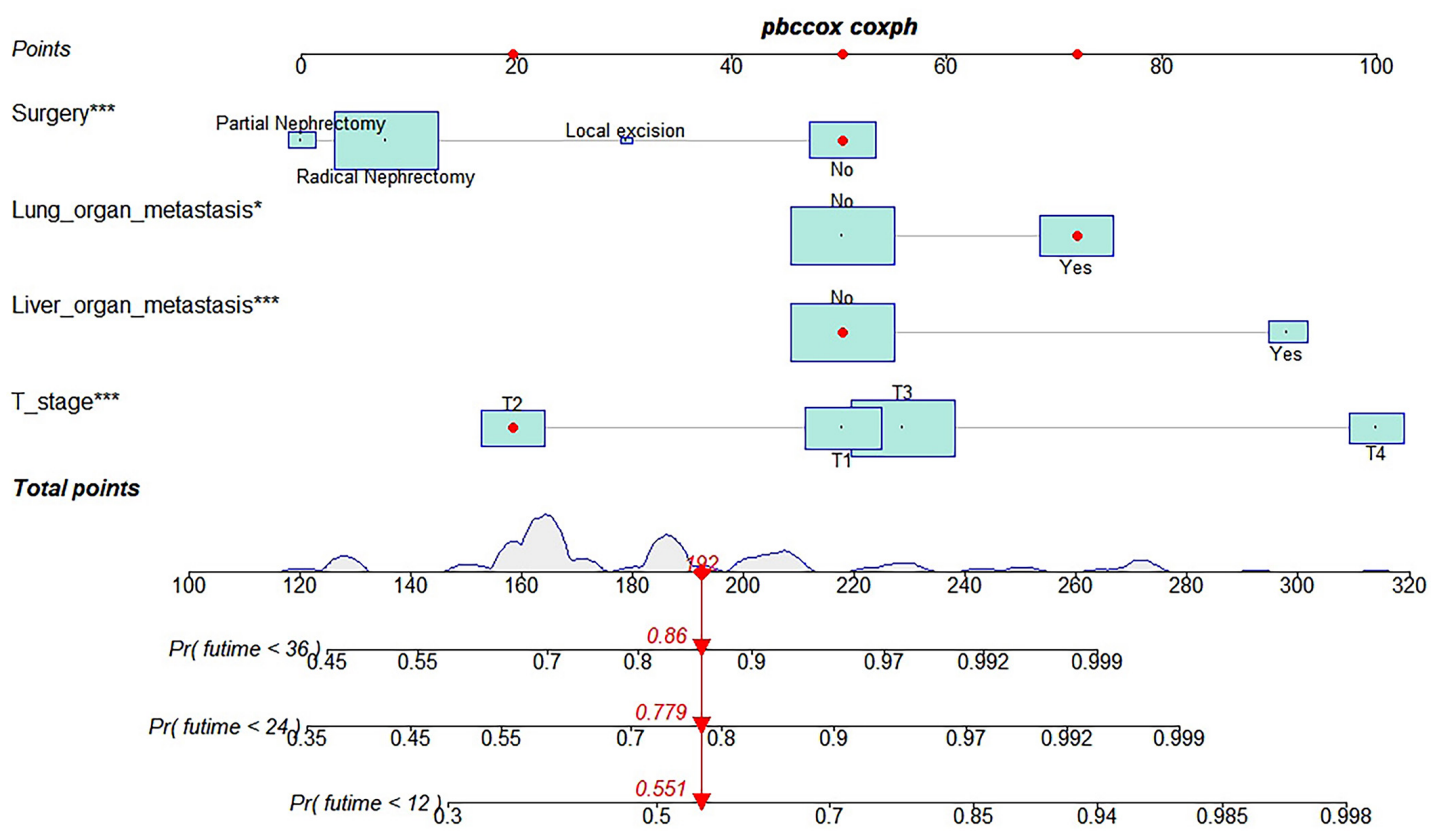
Prognostic nomogram in predicting 12-, 24-, and 36-months overall survival (OS) for elderly kidney cancer with bone metastasis (KCBM) patients. *** is the value of significance.

**Figure 8:**
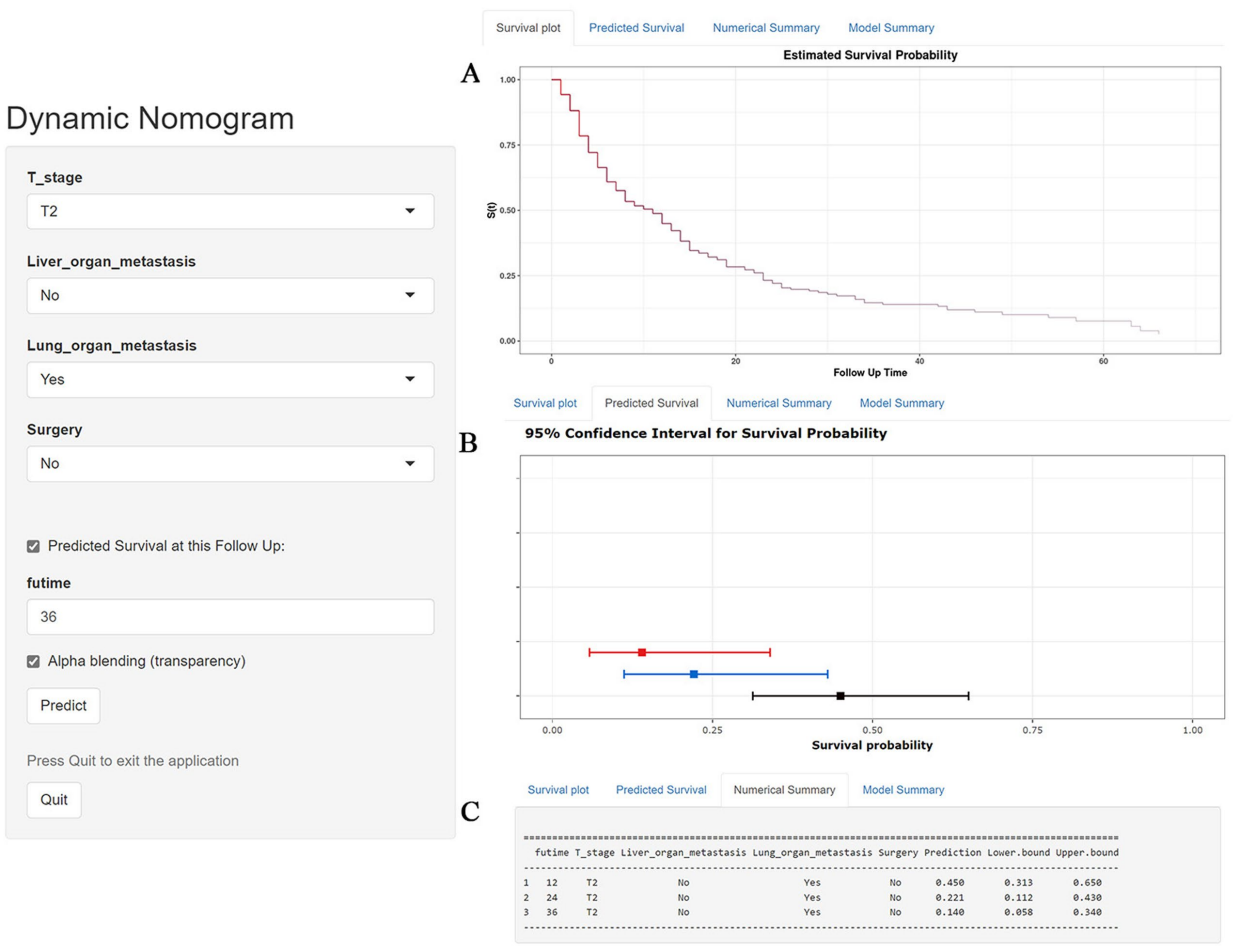
A web-based nomogram for predicting overall survival (OS) in an elderly kidney cancer with bone metastasis (KCBM) patient (T stage: T2, Liver metastasis: no, Lung metastasis: yes, Surgery: no). **(A)** The curve of the estimated probability of OS for this patient over time. **(B)** 95% confidence intervals of the 12-, 24-, and 36-month OS probabilities for this patient. **(C)** Numerical summary of the 12-, 24-, and 36-month OS probabilities for this patient. Due to a large number of visitors to the webpage, if the application cannot be used normally, please click “Quilt” or “Reload” in the lower-left corner to try again.

Table 3 showed the baseline data of elderly KCBM patients. Among the 17,404 elderly KC patients, a total of 394 patients developed BM, of which 297 patients (75.4%) opted for surgery, 197 patients (50.0%) for RT, and 214 patients (54.3%) for CT. The chi-square (× 2) test showed no statistical difference between the baseline variables of the different sets (*p* > 0.05; Table 3). As shown in Table 4, univariate Cox regression analysis indicated that patients with 8317/3 (chromophobe cell carcinoma), higher T stage (T3 and T4), N1 stage and brain/liver/lung metastasis may have a poorer prognosis (Table 4). Multivariate Cox regression analysis finally identified T stage, liver metastasis, lung metastasis and surgery as independent prognostic factors for elderly KCBM patients. Subsequently, we created the prognostic nomogram on elderly KCBM patients at 12, 24 and 36 months based on the independent prognostic factors described above (Figure 7), and an online version is accessible *via* https://prognostic.shinyapps.io/dynnomapp/ (Figure 8). The way to use the nomogram is to find the point on each variable axis that corresponds to the patient and then draw a line up to intersect the fraction axis above, which is the fraction corresponding to a single variable. The scores of the other variables were determined in the same way, and then the scores of all variables were summed to find the corresponding points on the total score axis below, and the point on the total score axis was used as the starting point to draw a line downward, and the intersect point at the OS probability axis of different times was the prognostic OS probability. For instance, an older KCBM patient who was more than 65 years, T2 stage, with lung metastasis, no liver metastasis, and did not undergo surgery. Lines are drawn upward to determine the points received by each variable; the sum of these points (192) is located on the total points axis, and a line is drawn downward to the OS axes to determine that the probability of OS at 12 months was 44.9% (Figure 7).

**Table 3:** The demographic and clinicopathological characteristics of elderly KC patients with BM.

Variables	Total set (*N* = 394,%)	Training set (*N* = 278,%)	Validation set (*N* = 116,%)	*p* value
Age				0.411
<70	172(43.7)	124(44.6)	48(41.4)	
70–80	189(48.0)	134(48.2)	55(47.4)	
>80	33(8.4)	20(7.2)	13(11.2)	
Sex				0.836
Female	143(36.3)	100(36.0)	43(37.1)	
Male	251(63.7)	178(64.0)	73(62.9)	
Race				0.268
Black	28(7.1)	19(6.8)	9(7.8)	
Other	20(5.1)	11(4.0)	9(7.8)	
White	346(87.8)	248(89.2)	98(84.5)	
Histological type				0.540
8120/3	23(5.8)	18(6.5)	5(4.3)	
8130/3	9(2.3)	9(3.2)	0(0.0)	
8260/3	12(3.0)	9(3.2)	3(2.6)	
8310/3	213(54.1)	148(53.2)	65(56.0)	
8312/3	74(18.8)	50(18.0)	24(20.7)	
8317/3	4(1.0)	3(1.1)	1(0.9)	
Other	59(15.0)	41(14.7)	18(15.5)	
Laterality				0.298
Left	196(49.7)	143(51.4)	53(45.7)	
Right	198(50.3)	135(48.6)	63(54.3)	
Tumor size				0.218
≤40 mm	43(10.9)	35(12.6)	8(6.9)	
40-80 mm	197(50.0)	134(48.2)	63(54.3)	
≥80 mm	154(39.1)	109(39.2)	45(38.8)	
Grade				0.442
Grade I	5(1.3)	2(0.7)	3(2.6)	
Grade II	87(22.1)	64(23.0)	23(19.8)	
Grade III	175(44.4)	122(43.9)	53(45.7)	
Grade IV	127(32.2)	90(32.4)	37(31.9)	
T stage				0.497
T1	100(25.4)	69(24.8)	31(26.7)	
T2	61(15.5)	48(17.3)	13(11.2)	
T3	185(47.0)	127(45.7)	58(50.0)	
T4	48(12.2)	34(12.2)	14(12.1)	
N stage				0.557
N0	284(72.1)	198(71.2)	86(74.1)	
N1	110(27.9)	80(28.8)	30(25.9)	
Brain metastasis				0.523
No	362(91.9)	257(92.4)	105(90.5)	
Yes	32(8.1)	21(7.6)	11(9.5)	
Liver metastasis				0.964
No	346(87.8)	244(87.8)	102(87.9)	
Yes	48(12.2)	34(12.2)	14(12.1)	
Lung metastasis				0.069
No	251(63.7)	185(66.5)	66(56.9)	
Yes	143(36.3)	93(33.5)	50(43.1)	
Surgery				0.119
No	97(24.6)	77(27.7)	20(17.2)	
Local excision	2(0.5)	2(0.7)	0(0.0)	
Partial nephrectomy	19(4.8)	13(4.7)	6(5.2)	
Radical nephrectomy	276(70.1)	186(66.9)	90(77.6)	
Radiotherapy				0.658
No	197(50.0)	137(49.3)	60(51.7)	
Yes	197(50.0)	141(50.7)	56(48.3)	
Chemotherapy				0.120
No	180(45.7)	120(43.2)	60(51.7)	
Yes	214(54.3)	158(56.8)	56(48.3)	

**Table 4:** Univariate and multivariate Cox regression analysis for identification independent prognostic factors in elderly KC patients with BM.

Variables	Univariate analysis			Multivariate analysis		
	HR	95%CIs	*p*-Value	HR	95%CIs	*p*-value
Age						
<70	Reference					
70–80	1.197	0.903–1.585	0.211			
>80	1.253	0.736–2.133	0.407			
Sex						
Female	Reference					
Male	0.988	0.746–1.309	0.935			
Race						
Black	Reference					
Other	1.194	0.530–2.689	0.669			
White	0.977	0.567–1.683	0.933			
Histological type						
8120/3	Reference			Reference		
8130/3	0.651	0.283–1.501	0.314	1.014	0.426–2.410	0.975
8260/3	0.588	0.245–1.411	0.234	1.177	0.472–2.938	0.727
8310/3	0.311	0.187–0.520	<0.001	0.604	0.339–1.075	0.087
8312/3	0.553	0.316–0.968	0.038	0.791	0.427–1.465	0.456
8317/3	0.452	0.105–1.954	0.288	0.785	0.177–3.489	0.750
Other	0.663	0.375–1.171	0.157	1.135	0.615–2.094	0.686
Laterality						
Left	Reference					
Right	1.138	0.869–1.491	0.347			
Tumor size						
<40 mm	Reference					
40-80 mm	0.897	0.591–1.362	0.611			
>80 mm	1.023	0.672–1.556	0.916			
Grade						
I	Reference					
II	0.370	0.089–1.535	0.171			
III	0.576	0.142–2.341	0.440			
IV	0.779	0.191–3.176	0.727			
T Stage						
T1	Reference			Reference		
T2	0.667	0.429–1.038	0.073	0.616	0.391–0.970	0.037
T3	1.074	0.764–1.512	0.680	1.005	0.686–1.473	0.980
T4	2.933	1.866–4.609	<0.001	1.848	1.135–3.009	0.013
N Stage						
N0	Reference			Reference		
N1	1.949	1.460–2.602	<0.001	1.301	0.944–1.794	0.108
Brain organ metastasis						
No	Reference					
Yes	1.427	0.890–2.288	0.140			
Liver organ metastasis						
No	Reference			Reference		
Yes	2.788	1.918–4.052	<0.001	1.642	1.072–2.514	0.023
Lung organ metastasis						
No	Reference			Reference		
Yes	1.571	1.185–2.084	0.002	1.382	1.021–1.871	0.036
Surgery						
No	Reference			Reference		
Local excision	1.251	0.306–5.119	0.755	0.733	0.165–3.257	0.684
Partial nephrectomy	0.318	0.152–0.663	0.002	0.439	0.206–0.934	0.032
Radical nephrectomy	0.477	0.355–0.641	<0.001	0.454	0.324–0.637	<0.001
Radiotherapy						
No	Reference					
Yes	1.005	0.767–1.316	0.972			
Chemotherapy						
No	Reference					
Yes	0.916	0.696–1.206	0.533			

**Figure 9:**
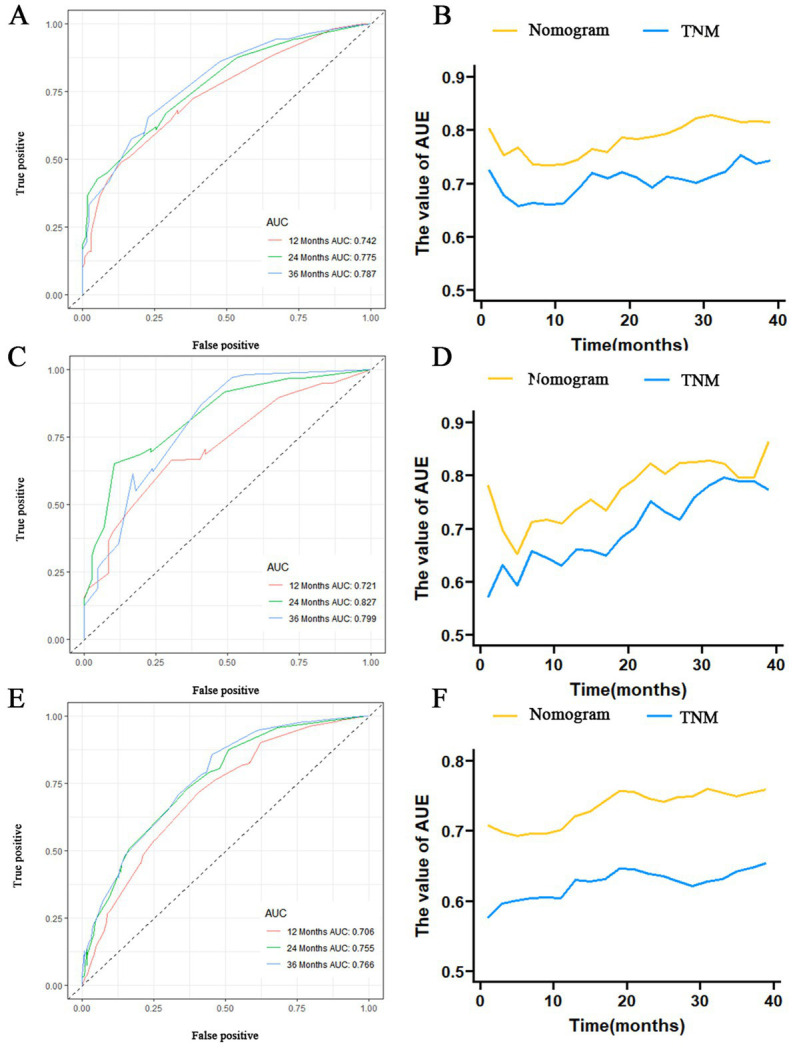
Receiver operating characteristic (ROC) curve analysis of nomograms at 12-, 24-, and 36-months in the training set **(A)**, validation set **(C)** and extended validation set **(E)**. Time-dependent ROC curves for comparing the discriminatory ability between nomograms and TNM staging system in the training set **(B)**, validation set **(D)** and extended validation set **(F)**.

**Figure 10:**
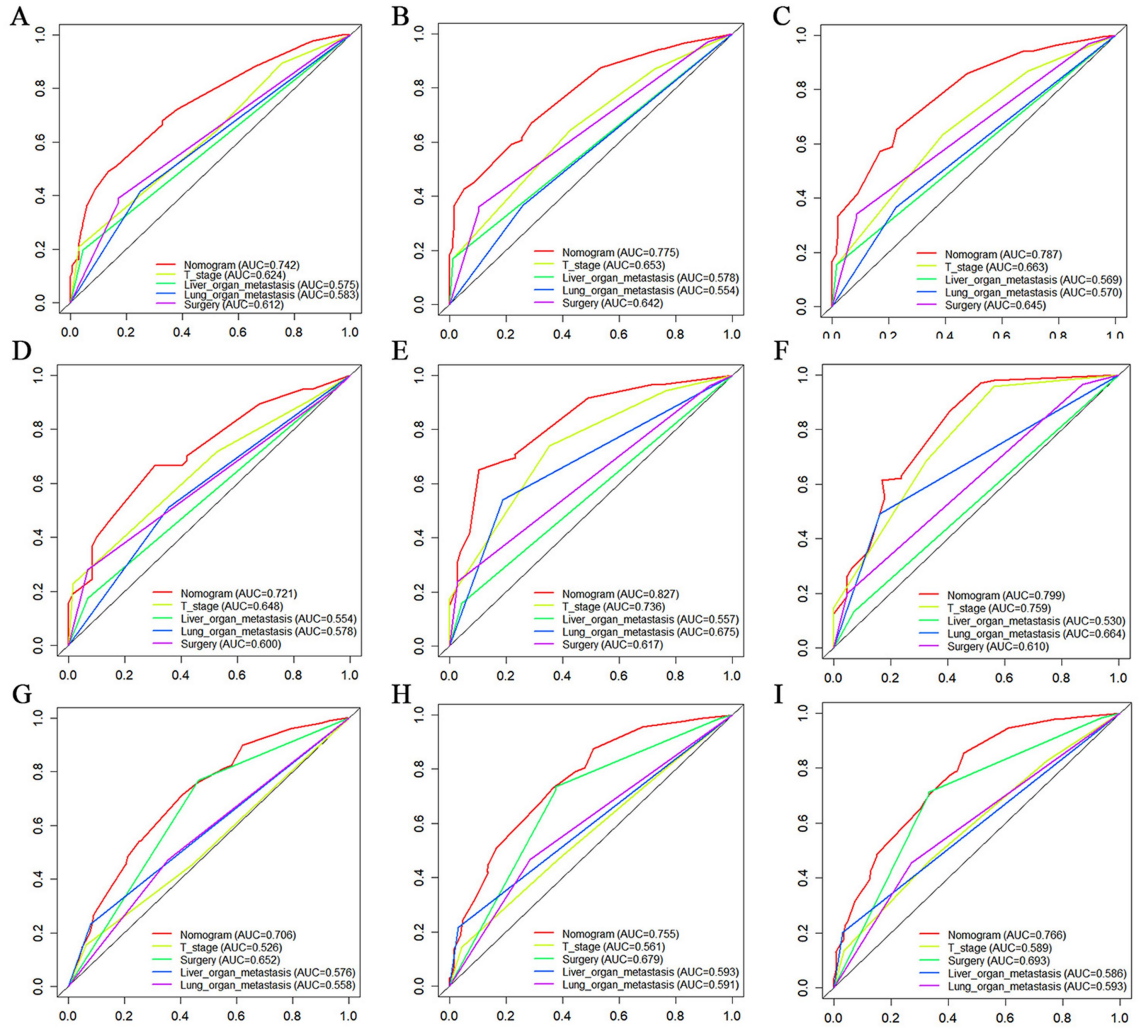
The Comparative Receiver operating characteristic (ROC) for nomogram and single independent prognostic factors at 12 months, 24 months and 36 months in the training set **(A–C)**, validation set **(D–F)** and extended validation set **(G–I)**.

**Figure 11:**
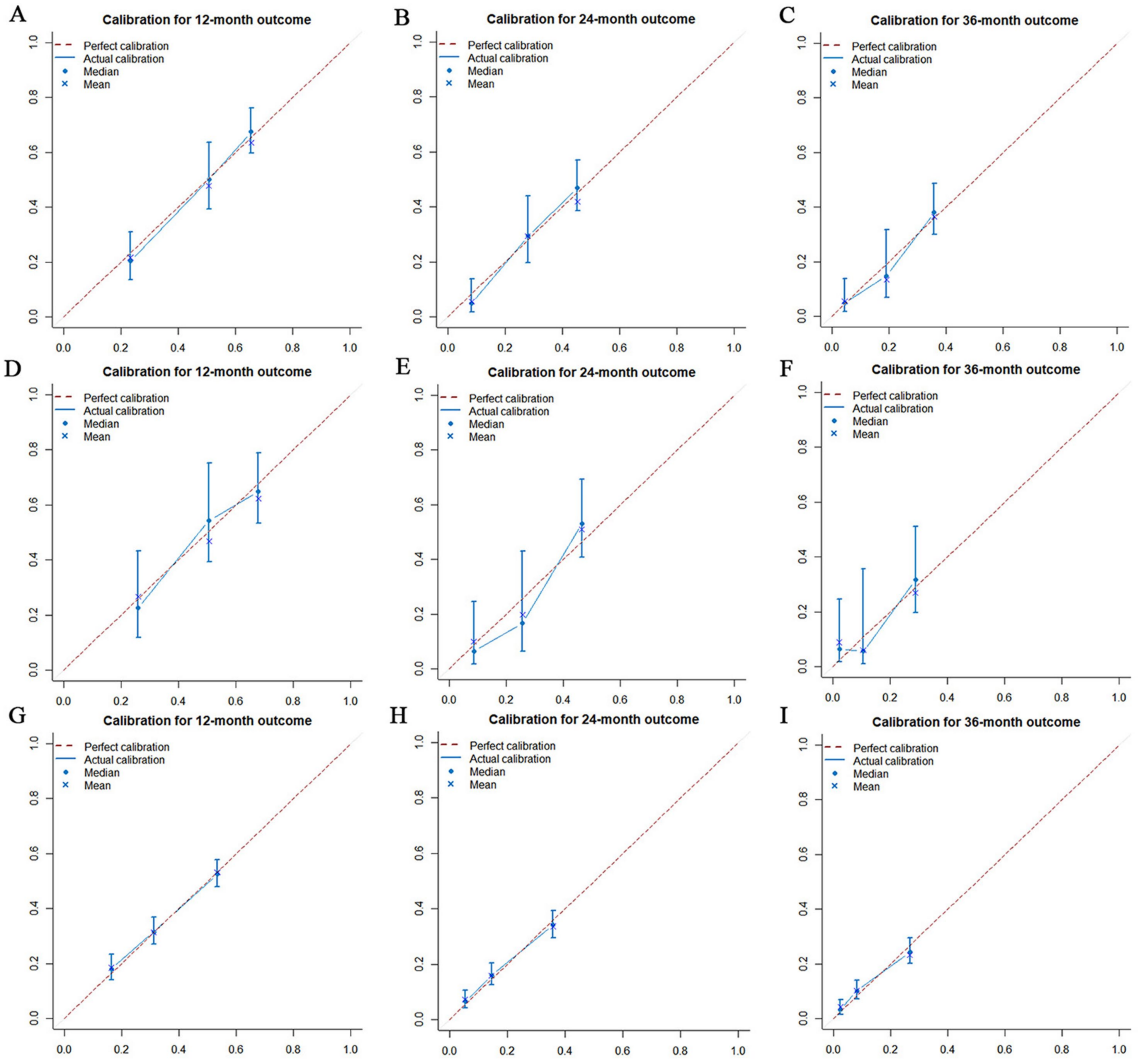
Calibration curves for overall survival (OS) at 12 months **(A)**, 24 months **(B)** and 36 months **(C)** in the training set, Calibration curves for OS at 12 months **(D)**, 24 months **(E)** and 36 months **(F)** in the validation set and Calibration curves for OS at 12 months **(G)**, 24 months **(H)** and 36 months **(I)** in the extended validation set.

**Figure 12:**
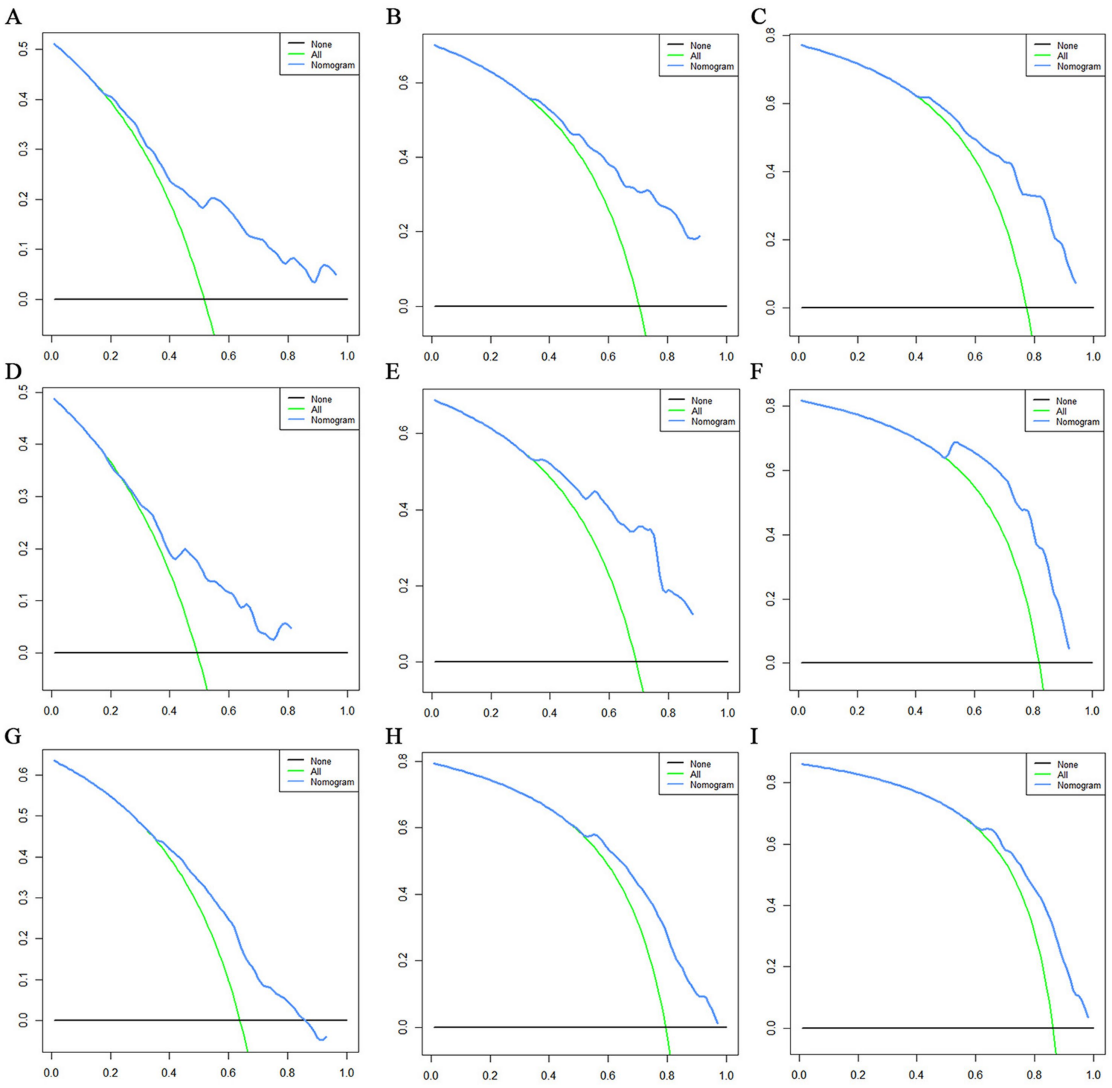
Decision curve analysis (DCA) curves for overall survival (OS) at 12 months **(A)**, 24 months **(B)** and 36 months **(C)** in the training set, DCA curves for OS at 12 months **(D)**, 24 months **(E)** and 36 months **(F)** in the validation set and DCA curves for OS at 12 months **(G)**, 24 months **(H)** and 36 months **(I)** in the extended validation set.

**Figure 13:**
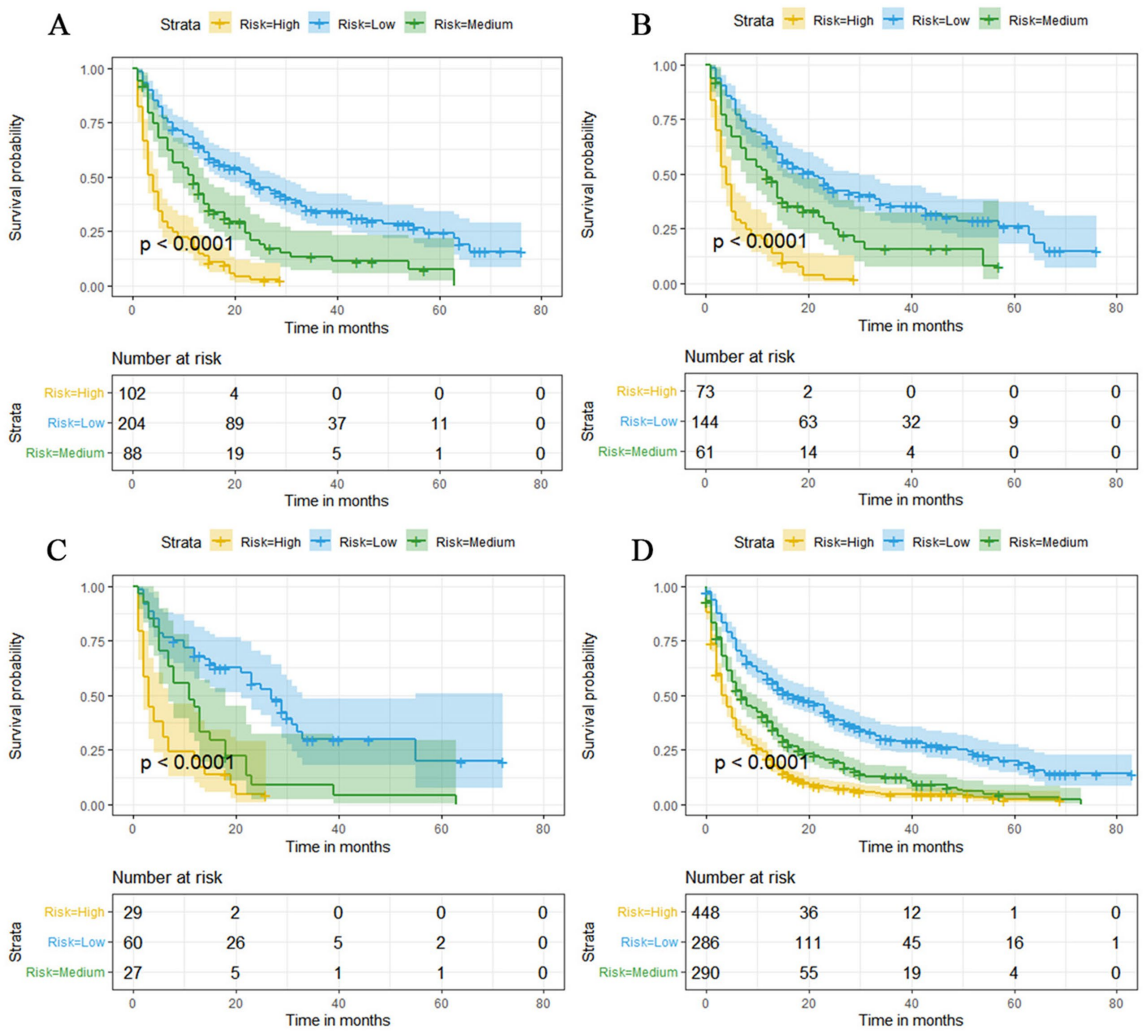
Kaplan–Meier survival curves for three high, medium and low mortality risk subgroups in the overall elderly kidney cancer with bone metastasis (KCBM) set **(A)**, training set **(B)**, validation set **(C)** and extended validation set **(D)**.

The prognostic nomogram showed that T-stage has the greatest impact on OS, followed by surgery. Following, the ROCs of the training and validation sets were analyzed to assess the predictive efficacy of the prognostic nomogram. The corresponding AUC values at 12, 24 and 36 months were: training set: 0.742, 0.775 and 0.787 (Figure 9A); validation set: 0.721, 0.827 and 0.799 (Figure 9C). It showed that the prognostic nomogram has good predictive performance. Meanwhile, we also compared the predictive efficacy of nomograms and single independent prognostic factors at different time points. The ROC and AUC of the training set (Figures 10A–C) and validation set (Figures 10D–F) at 12-, 24- and 36-moths both demonstrated that the prognostic nomogram has better predictive efficacy than single independent prognostic factors. Furthermore, time-dependent ROC showed that the predictive efficacy of the nomogram was better than that of the existing AJCC TNM staging system (Figures 9B,D). In addition, the calibration curves of training set (Figures 11A–C) and validation set (Figures 11D–F) showed good concordance between the predicted OS of the prognostic nomogram and the clinically observed OS. DCA of training set (Figures 12A–C) and validation set (Figures 12D–F) showed that the prognostic nomogram had a significant net benefit. Later, we went back to the database and rescreened 1,024 elderly KCBM patients through the identified independent prognostic factors as an extended validation set. The ROC (the AUCs at 12, 24, 36 months: 0.707, 0.768, 0.779), time-dependent ROC, the Comparative ROC for nomogram and single independent prognostic factors, calibration curves and DCA of the extended validation set all reconfirmed the good predictive efficacy and clinical utility of our newly constructed prognostic nomogram (Figures 9E,F, 10G–I, 11G–I, 12G–I). Finally, we calculated the prognostic score for each elderly KCBM patient by using the constructed prognostic nomogram. All elderly KCBM patients were divided into low (risk score: <186), medium (risk score: 186–200) and high (risk score: >200) risk groups according to optimal cutoff values determined by X-tile software. K–M survival analysis demonstrated significant differences among the three risk groups (*p* < 0.0001) in the overall elderly KCBM cohort (Figure 13A), training sets (Figure 13B), validation sets (Figure 13C) and extended validation sets (Figure 13D) indicating the validity of the nomogram-based risk stratification system.

## Discussion

With the advent of aging society, the number of elderly people will gradually increase, which means that the number of elderly KC patients will also increase further (23). Moreover, elderly people are the main group of patients with metastatic KC, which may be related to the high heterogeneity of KC as well as the deterioration of physical function and immune resistance of the elderly (24). Therefore, the diagnosis and treatment of the elderly KC patients should be given a higher attention. In our study, age, histological type, tumor size, grade, T stage, N stage, and brain/liver/lung metastasis were important risk factors for BM in elderly KC patients. In elderly KC patients, the risk of BM was highest in the 65–70 years age group, while the risk of bone metastasis decreased above 70 years of age, which may be related to the fact that capillary sclerosis weakened the spread of cancer cells (25). Among the histological types, the highest risk of BM was found in Renal cell carcinoma (8,312/3) and Clear cell adenocarcinoma (8,310/3). This is in line with previous studies. Dong S et al. (26) investigated the risk factors for developing BM in KC patients and constructed prediction models aimed at providing accurate prediction of BM risk in KC patients. Their results showed that the most likely histological types for BM were Renal cell carcinoma (8,312/3) and Clear cell adenocarcinoma (8,310/3). In addition, Yan F et al. (27) found that migration and invasion inhibitory protein (MIIP), which inhibits cancer cell proliferation and angiogenesis, was significantly downregulated in patients with clear cell adenocarcinoma, which also provides an explanation for the susceptibility of clear cell adenocarcinoma to metastasis (27).

The impact of tumor characteristics on prognosis is usually significant, which has been confirmed in many previous studies (26, 28). Our findings suggested that elderly KC patients with larger and less differentiated tumors are more prone to BM. This may be due to the fact that larger tumors mean that more tissues are likely to be invaded and less differentiated tumors mean more bio-aggressiveness, both of which may increase the probability of tumor metastasis (29). Furthermore, T-stage and N-stage have been shown to be independent risk factors for the development of BM in elderly KC patients. In the current TNM staging system of AJCC, T means the size and depth of tumor infiltration, and N means the site and number of lymphatic metastases. Higher T stage and N stage indicate that the primary tumor has developed deep infiltration and a certain number of lymphatic metastases, which inevitably increases the possibility of further BM (30, 31). Finally, brain/liver/lung metastasis were also confirmed by our study as significant risk factors for BM. This may be due to the presence of metastases to vital organs at the initial diagnosis of the KC, indicating that cancer cells have escaped *via* the vascular or lymphatic system or other forms of metastasis, which makes the probability of further BM much higher (32, 33).

In addition, surgery, T stage, liver metastasis and lung metastasis were shown to be significantly associated with the prognosis of elderly KCBM patients. T stage often affect the prognosis of survival in elderly KCBM patients, which is understandable because higher T stage and may mean more vascular and tissue invasion. As time progresses, the division of tumor cells would get further out of control (34). All of these might promote the development of metastatic disease. For elderly KCBM patients, the time-dependent ROC results showed that the prognostic nomogram we constructed has more obvious advantages compared with the AJCC TNM staging system (Figures 9B,D,F). Moreover, Zhou et al. (35) found that KCBM patients with liver or lung metastasis at the time of initial diagnosis had a significantly increased risk of death. This is consistent with our findings, which similarly showed that liver metastasis (HR: 1.557, 95%CI: 1.097–2.211, *p* < 0.05) and lung metastasis (HR: 1.432, 95%CI: 1.117–1.836, *p* < 0.05) were important factors influencing the prognosis of elderly KCBM patients. A reasonable explanation was that multisite metastasis leads to a more complex alteration of the internal microenvironment, with stronger anti-tumor immunity and a more severe inflammatory response resulting in stronger potential resistance to tumor cells that have colonized other sites (36).

At present, the main treatment modality for elderly KCBM patients is surgery (37).

Bone metastases (BM) in KC patients are usually isolated and are easily resistant to RT and CT, so surgical excision becomes the mainstay of treatment (38). Our study found that partial nephrectomy (HR: 0.400, 95% CI: 0.208–0.770, *p* < 0.05) and radical nephrectomy (HR: 0.468, 95% CI: 0.350–0.625, *p* < 0.05) had the best prognosis for elderly KCBM patients. Similar findings have been reported in previous studies. Ratasvuori M et al. (39) found a fourfold increase in prognostic survival in elderly KCBM patients who underwent surgery compared to those who did not. Although surgical treatment is currently providing a better survival benefit for elderly KCBM patients, surgery may increase the incidence of renal insufficiency due to the often co-morbidities and poorer physiological compensations in the elderly, thus reducing prognostic OS after surgery (40). Consequently, we suggest that a more comprehensive assessment and personalized clinical management program should be provided in the diagnosis and treatment of elderly KCBM patients.

Nevertheless, there are several limitations of this study. Firstly, this was a retrospective study and we could not avoid selection bias. Secondly, the SEER database does not record genetic information (gene expression and chromosomal alterations) and co-morbidities (hypertension, immune-related diseases and diabetes) of the patients, which may have an impact on the results (41, 42). Finally, due to the low prevalence of KCBM, it is difficult to collect a sufficient number of external validation cohorts in the same region to externally validate our constructed prediction models; therefore, prospective studies are needed for further validation.

## Conclusion

This study retrospectively investigated the risk factors for the development of BM in elderly KC and the prognostic factors after BM. Based on these factors, nomograms were constructed to assist surgeons in identifying patients at high risk for BM and in assessing overall survival after BM. The web-based nomograms and mortality risk stratification system provide significant considerations for multidisciplinary management.

## Data availability statement

The original contributions presented in the study are included in the article/supplementary material, further inquiries can be directed to the corresponding author.

## Author contributions

LJ and DZ conceived and designed the study and revised the manuscript. LJ and YT collected the clinical data and literature review. LJ and JJ conducted the statistical analysis. LJ generated the figures and tables, and wrote the manuscript. DZ supervised the research. LJ, YT, JJ, and DZ critically read the manuscript to improve intellectual content. All authors contributed to the article and approved the submitted version.

## Conflict of interest

The authors declare that the research was conducted in the absence of any commercial or financial relationships that could be construed as a potential conflict of interest.

## Publisher’s note

All claims expressed in this article are solely those of the authors and do not necessarily represent those of their affiliated organizations, or those of the publisher, the editors and the reviewers. Any product that may be evaluated in this article, or claim that may be made by its manufacturer, is not guaranteed or endorsed by the publisher.

## Footnotes

^1^ www.seer.cancer.gov

## Abbreviations

KC: kidney cancer
BM: bone metastasis
KCBM: kidney cancer with bone metastasis
SEER: surveillance, epidemiology and end results
ICD-O-3: International Classification of Diseases for Oncology, 3rd Edition
K-M: Kaplan–Meier
OS: overall survival
CSS: cancer specific survival
AJCC: American Joint Committee on Cancer
CI: confidence interval
OR: odd ratio
HR: hazard ratio
ROC: receiver operating characteristic
AUC: area under curve
DCA: decision curve analysis
